# Polyphenol Mechanisms against Gastric Cancer and Their Interactions with Gut Microbiota: A Review

**DOI:** 10.3390/curroncol29080417

**Published:** 2022-07-25

**Authors:** Matu Li, Ya Zheng, Jinyu Zhao, Meimei Liu, Xiaochuang Shu, Qiang Li, Yuping Wang, Yongning Zhou

**Affiliations:** 1 The First Clinical Medical School, Lanzhou University, Lanzhou 730000, China; limt20@lzu.edu.cn (M.L.); zhaojy2021@lzu.edu.cn (J.Z.); liumm20@lzu.edu.cn (M.L.); 2 Department of Gastroenterology, The First Hospital of Lanzhou University, Lanzhou 730000, China; zhengy2010@lzu.edu.cn (Y.Z.); shuxiaochuang@aliyun.com (X.S.); liqldyy@126.com (Q.L.); 3 Key Laboratory for Gastrointestinal Diseases of Gansu Province, The First Hospital of Lanzhou University, Lanzhou 730000, China; 4 Department of General Surgery, The First Hospital of Lanzhou University, Lanzhou 730000, China

**Keywords:** polyphenols, gastric cancer, intestinal flora, mechanism, interaction

## Abstract

The lack of new drugs and resistance to existing drugs are serious problems in gastric cancer(GC) treatment. The research found polyphenols possess anti-*Helicobacter pylori(Hp)* and antitumor activities and may be used in the research and development of drugs for cancer prevention and treatment. However, polyphenols are affected by their chemical structures and physical properties, which leads to relatively low bioavailability and bioactivity in vivo. The intestinal flora can improve the absorption, utilization, and biological activity of polyphenols, whereas polyphenol compounds can increase the richness of the intestinal flora, reduce the activity of carcinogenic bacteria, stabilize the proportion of core flora, and maintain homeostasis of the intestinal microenvironment. Our review summarizes the gastrointestinal flora-mediated mechanisms of polyphenol against GC.

## 1. Introduction

According to the Global Cancer Epidemiology Statistics (GLOBOCAN) in 2020 worldwide [1], gastric cancer (GC) remains important cancer worldwide and is responsible for over one million new cases and an estimated 769,000 deaths (equating to 1 in every 13 deaths globally), ranking fifth for incidence and fourth for mortality globally. Considering the new cases of and deaths due to GC, China ranks third worldwide. According to data from China [2], the five-year survival rate for most early GCs after radical endoscopic therapy is more than 90, but the 5-year survival rate is still less than 30% even after comprehensive treatment-based surgery [3]. Achieving early diagnosis and treatment is a key part of the global GC prevention and control work. However, most areas with a high incidence of GC in the world still lack a mature prevention and control system for gastric cancer. More than 50% of the patients were at the advanced stage of GC at the time of initial diagnosis, so they lost the chance of radical surgery, and thus, they could only adopt a comprehensive treatment scheme based on anti-tumor drugs [4]. A recent meta-analysis showed that third-line therapy (TLT) is effective and safe in the treatment of advanced or metastatic GC, such as overall survival (OS), progression-free survival (PFS) and disease control rate (DCR) [5]. Chemotherapy, in the dominant position, has a relatively wide application scope. Fluorouracil plus platinum is the first line regimen, but the benefit of chemotherapy alone is limited, and the Median Survival Time (MS) is only 8 months, so chemotherapy is recommended combined with targeted therapy [6]. Targeted therapy is in targeting human epidermal growth factor receptor-2 (HER-2), vascular endothelial growth factor receptor (VEGF), tyrosine kinase inhibitor (TKI), and so on [7]. As early as 2010, the ToGA trial established the first-line treatment for patients with advanced GC who were HER-2 positive with trastuzumab combined with chemotherapy [8]. With the development of research on HER-2-targeted therapy for advanced GC, in January 2021, the Food and Drug Administration (FDA) of the United States approved trastuzumab deruxtecan (T-Dxd) for the treatment of unresectable, locally advanced, or metastatic GC, which previously received a trastuzumab regimen, thus further perfecting the targeted treatment of GC. However, the population suitable for targeted therapy is relatively limited, and the therapeutic effect is also different among individuals [9]. With the in-depth study of the tumor immune microenvironment (TME), the efficacy of immunotherapy represented by immune checkpoint inhibitors (ICIs) has been clear, especially in GC, with high expression of programmed death protein-1 (PD-1), programmed death ligand-1 (PD-L1), EB virus infection (EBV) and microsatellite instability (MSI), where the curative effect of ICIs is the most significant. The therapeutic effects of genomic stable type (GS) and chromosome unstable type need to be further studied [10]. However, ICIs work only against specific immune checkpoints (cell-surface molecules) and are almost ineffective in patients with low immune checkpoint expression levels [11]. However, with the continuous improvement in antineoplastic drug treatment for GC, the problem of tumor drug resistance has obviously not been improved [12]. Therefore, development of new drugs and complimentary medicine is essential, and plant polyphenols have been reported to have a good anti-cancer effect, which has attracted the wide attention of researchers.

Polyphenols are secondary metabolites from plants, widely present in foods and beverages with plant origins (e.g., fruits, vegetables, grains, soy, tea, and wine) [13]. Results of epidemiological research and meta-analyses implied that a polyphenol-rich diet has a protective effect against tumor, cardiovascular disease, diabetes mellitus, osteoporosis, and neurodegenerative diseases [14,15]. Polyphenols and polyphenol subclasses intake may reduce GC risk [16]. In addition, several literature findings have suggested that dietary polyphenols inhibit proliferation, induce apoptosis and reduce drug resistance in GC cells [17]. However, polyphenols’ function is affected by many factors, both intrinsic and extrinsic. For example, the gastrointestinal flora plays significant roles in the process of polyphenol absorption and metabolism. Most natural polyphenolic compounds must be absorbed and utilized under the action of specific intestinal flora, and phenolic metabolites can have activities that are not found in the original compounds [18]. On the contrary, polyphenols have a regulatory effect on the intestinal flora. They function as prebiotics by providing substrates required for microbial metabolism and interacting with microbial-related enzymes, enhancing beneficial flora growth, inhibiting carcinogenic flora proliferation, and maintaining the homeostasis of the intestinal microenvironment [19]. Therefore, our review concentrates on the anti-GC mechanisms of polyphenols mediated by gastrointestinal flora.

## 2. Polyphenol Anti-GC Mechanism

Previous studies have indicated polyphenols’ chemopreventive effect as antioxidant, antiproliferative, antibacterial, apoptosis-promoting compounds, and their role in regulating signaling pathways that prevent or reverse tumor differentiation. This includes two main aspects: polyphenols directly inhibit the occurrence of GC, and polyphenols eliminate GC risk factors, such as the infection by *Helicobacter pylori (Hp).*

### 2.1. Direct Protective Effect of Polyphenols 

#### 2.1.1. Polyphenols Protect against DNA Damage

Polyphenols have the same average reduction potential as vitamin E and are considered to be the richest antioxidants in the daily diet [20,21]. The biological activity depends on chemical structure, including the hydroxylation position of a single compound and the substitution of specific hydroxyl groups. The presence of hydroxyl groups makes polyphenols excellent hydrogen-bond donors [22]. Polyphenols have a high affinity for proteins and DNA, which promotes antioxidant properties and anti-free radical-mediated anti-DNA damage effects [23]. For example, curcumin inhibits GC growth by generating many reactive oxygen species, leading to the depletion of mitochondrial DNA content and DNA polymerase, altering the bioenergetics of the cells [24]. The mechanism is mainly regulated by the p53-p21/Gadd45a cyclin/CDK Rb/E2f-dnmt1 axis in damaged DNA repair [24]. Furthermore, studies have shown that curcumin analogs target topoisomerase II in human cancer cells, thus directly blocking the activity of topoisomerase II, and the chain in the DNA chain cannot be reconnected, leading to cancer cell apoptosis [25]. However, a Peng et al. study showed that polyphenols seemed to only have an antioxidant effect but did not repair the oxidized cells. This conclusion needs to be confirmed by more studies [26].

#### 2.1.2. Apoptosis of Tumor Cells Induced by Polyphenols

Polyphenols have great potential for cancer prevention through the induction of apoptosis [27]. Natural polyphenols promote GC cell apoptosis by regulating target kinases. Researchers found that terminal ascorbic acids can activate the p38 MAPK-c-Jun-terminal kinase (JNK) pathway and promote apoptosis of GC cells [28]. Otherwise, phenolic compounds in the Begonia fruit extract inhibit the tumor cells’ growth, mainly by increasing the expression of Bcl-2 and Bcl-xL, and inhibiting Bax and Bak expression [29]. Similarly, curcumin significantly downregulate the expression level of Bcl-2, CDK4, and cyclin D1 in cells and tissues, thereby inhibiting SGC-7901 GC cell proliferation and inducing cell apoptosis [30]. It also regulates the proliferation, autophagy, and apoptosis of GC cells by affecting the PI3K and p53 signaling pathways [31]. Kaempferol activates IRE1-JNK-CHOP signaling pathway from cytoplasm to nucleus and inhibits epigenetic changes mediated by G9a (HDAC/G9a axis), thus activating autophagic death of GC cells [32]. Pectolina rigenin may lead to cell cycle arrest, autophagy and apoptosis in G2/M phase of GC cells by downregulating PI3K/AKT/mTOR pathway [33]. Resveratrol promotes cell apoptosis and against proliferation by combining with PIM-1 and inhibiting its catalytic activity [34]. The anti-apoptosis effect of polyphenols may be related to the inhibition of the activation of NF-κB involved in Notch and Wnt pathways [35]. Ho et al. indicated that gallic acid’s inhibitory effect on GC cells might connect with the NF-κB activity [36]. Curcumin inhibits the growth and promotes apoptosis of GC cells by the Wnt/-catenin pathway [37].

#### 2.1.3. Tumor Metastasis Inhibition and Invasion

Epithelial-mesenchymal transition (EMT) is vital for tumor cells to achieve metastatic ability and invasiveness. After EMT, patients with GC are more likely to develop resistance to various therapeutic drugs, which worsens their clinical outcomes. For example, resveratrol regulates EMT by interfering with the hedgehog pathway inhibiting the GC invasion and metastasis [38]. In addition, resveratrol can regulate the PTEN/Akt pathway to inhibit EMT of GC cells [39]. Lignin-like sauchinone downregulate the PI3K/Akt and Smad2/3 pathways to prevent TGF-β1-relevant EMT [40]. Luteolin reverses EMT and inhibits GC progression by restraining the Notch pathway [41]. Plant polyphenols reduce tumor metastasis and invasion by regulating the EMT pathway. Recent studies have shown that resveratrol may also prevent IL-6-induced GC metastasis through downregulating the activation of the Raf/MAPK pathway [42]. Pagliara et al. reported that the lemon peel polyphenol extract inhibits the invasiveness of GC cells by decreasing the MM9/2 expression level [43]. Polyphenolic compounds inhibit tumor metastasis and invasion through other mechanisms. For example, curcumin may inhibit liver metastasis in primary GC by inhibiting the CD1/CXCR4-related pathway [44] and HMGB1/VEGF-D pathway GC [45].

#### 2.1.4. Tumor Metastasis Inhibition and Invasion

Chemotherapeutic drug resistance has become a problem in GC. Studies have reported that compared with simple chemotherapy drug treatment group, the polyphenol-containing drug combined with chemotherapy increased the effect of GC chemotherapy [46]. The combination of oxaliplatin and rutin reduces the toxicity effects of chemotherapeutics and improves chemotherapy effect; the combination of luteolin and oxaliplatin can change the cell cycle ratio of SGC-7901 cells [47]. Baicalein promotes apoptosis and autophagy of GC cells through the Akt/mTOR and Nrf2/keap 1 pathway to improve sensitivity to cisplatin, and its effect is more intense than that of cisplatin or baicalein alone [48]. Similarly, cisplatin combined with avicularin significantly induces tumor cell apoptosis and reduces proliferation [17]. Cisplatin and resveratrol synergize the antitumor effect through endoplasmic reticulum stress-induced apoptosis and G2/M phase arrest [49]. The concentration and expression of angiogenesis-related factors are significantly downregulated after the combined treatment of quercetin and irinotecan, which may enhance the curative effect of irinotecan on the human GC cells [50]. Troxerutin inhibits STAT3/NF- B and Bcl-2 pathways to enhance the therapeutic function of 5- fluorouracil (5-FU) on GC [51]. The combination of 5-FU and catechin shows a better cytotoxic effect on tumor cells, and promotes the apoptosis of GC cells through reactive oxygen species [52]. Flavonoids can promote autophagy, inhibit EMT, block cell cycle and target ERK1/2/MAP pathway, showing selective anti-proliferation activity of adriamycin-resistant GC cells [53]. Rosmarinic acid combined with targeted therapy for GC has an excellent anticancer effect [54]. In addition, some polyphenol compounds, such as flavonoid polyphenols, have shown a more substantial anticancer effect than chemotherapeutic drugs [48]. Studies have shown that silibinin has significant cytotoxic activity on gastric adenocarcinoma cells (CI50: 60.17 ± 0.95 μg/mL) with a higher selectivity index compared with cisplatin. After metabolization silibinin showed an increase of cytotoxicity with a CI50 six-fold decrease (10.46 ± 0.25) [55].

## 3. Polyphenols Protect Indirectly from GC by Inhibiting *Hp*

*Hp* is considered the most critical member of the gastric microbiota, and its infection is a risk bacterium factor for GC [56]. Therefore, eradication of the infection is important for GC prevention and treatment. Because *Hp* can invade and colonize the gastric mucosa, its eradication has become a problem worldwide, but most antibiotics are not active in the acidic gastric environment. Therefore, new antibacterial compounds are actively being explored. Natural polyphenols and their secondary metabolites inhibit *Hp* activity. Based on *Hp* pathogenic factors, the mechanism of action of polyphenols against the bacterium mainly includes the following.

### 3.1. Restriction of Hp Colonization through Urease Inhibition

Urease is considered as one of the virulence factors of *Hp* and a necessary condition for infection. The apple polyphenol improves the chronic gastrointestinal effects caused by *Hp* through inhibiting urease effect [57]. Paulo et al. reported that resveratrol and red wine inhibit the growth of *Hp* through downregulating urease activity [58]. Procyanidins also have inhibitory effect against *Hp* urease, which is significantly related to the molecular size of procyanidins [59].

### 3.2. Inhibitory Effect of Bacterial Sialic Acid-Specific Adhesin and Downregulation on Expression of Inducible Cytidine Deaminase

*Hp* is parasitic on the human gastric mucosa and causes inflammation, atrophic gastritis, and GC. Adhesion is an essential component of the pathogen invasion and is a key event in the establishment of infection. Studies have indicated that polyphenols decrease the adhesion between *Hp* and the gastric mucosa, reduce the *Hp*-related inflammatory response, and reduce the incidence of *Hp*-associated GC. Additionally, 3.9 g/mL flavonoids inhibit approximately 90% of *Hp* growth by inhibiting adhesion [60]. In addition, in *Hp*-infected gastric epithelial cells, the activation of NF-κB can promote the abnormal expression of inducible cytidine deaminase, which is considered as one of the key mechanisms of *Hp* -related GC. Therefore, the inhibitory effect of NF-κB downregulates AID expression and plays a protective role. Curcumin may downregulate AID induced by inhibiting the NF-κB pathway and combat *Hp*-related gastric carcinogenesis [61].

### 3.3. Inhibition of the Release of Inflammatory Cytokines

IL-8 is the key cytokines involved in *Hp*-related inflammatory response. Torres et al. synthesized epicatechin semisynthetic derivatives from avocado peel and observed their adhesion to human GC cells and the induction of the proinflammatory release of IL-8 [62]. The study found that at 700 g/mL, the *Hp* adhesion rate to human stomach adenocarcinoma cells was less than 20% The production rate of IL-8 was less than 10%, indicating that epicatechin has anti-inflammatory functions on *Hp*-infected GC. The resveratrol pretreatment significantly inhibits *Hp*-induced IL-8 secretion and reactive oxygen species production [61]. The inhibitory function of resveratrol and epicatechin on IL-8 is probably related to their inhibitory activity on the NF-κB pathway, which downregulates the expression level of IL-8. Research has found that Walnut polyphenol extracts prevent *Hp*-related tumor growth by inhibiting STAT3 phosphorylation and nuclear translocation in gastric mucosal cells [63], and reversing precancerous atrophic gastritis [64]. Nobiletin has been confirmed to inhibit *Hp* infection and prevent *Hp*-mediated GC [65]. Notably, silymarin has 100% inhibitory effect on cytokines and NO related to *Hp* infection [55].

### 3.4. Inhibition of the Cytotoxic Activities of Hp Vacuolar Protein A (Vac A) and Cytotoxic Associated Protein A (Cag A)

Vac A and Cag A have important impacts in *Hp* pathogenesis. Infection with Vac A+ strain leads to vacuolization and apoptosis, whereas Cag A+ strain infection leads to severe gastritis and GC. Kaempferol plays anti-inflammatory and anti-cancer roles by decreasing the translocation of Cag A and Vac A [66]. In addition, the degree of gastric damage is quantitatively determined in mice, which tips that high-molecular-weight catechin-polymerized hop bud leaf extract (HBT) inhibits the activity of Vac A in vivo. Additionally, HBT can inhibit the activity and absorption of Vac A, while inhibiting Vac A-induced vacuolation of sensitive cells to inhibit the occurrence of gastric ulcer and inflammation [67]. Black rice extract, with anthocyanin as the main component, can also impede mRNA and protein levels of Cag A and Vac A [68]. Mahady et al. proved that resveratrol inhibits the Cag A+ *Hp* growth [69]. Animal studies have further confirmed that polyphenols limit damage to the gastric epithelium in mice model infected with *Hp* or treated by Vac A toxin [70]. Similarly, curcumin has an obvious inhibitory function on the activity of Cag A+ Hp [71]. All in all, polyphenols exert a variety of biological activities inhibiting the appearance of GC directly and indirectly, and have a protective impact by regulating gastrointestinal flora, such as *Hp*. Indeed, there is an extremely close relationship between polyphenols and intestinal microbes, which is closely related to the occurrence of GC. Importantly, gastrointestinal flora involves in the complete metabolic process of polyphenols, which significantly improves the absorption, utilization, and biological activity of polyphenols. In addition, polyphenols have a strong regulatory effect on the intestinal flora, thereby triggering an increase in the body beneficial bacteria to prevent GC occurrence.

## 4. Intestinal Flora Promotes the Transformation and Absorption of Polyphenols and Regulates Their Biological Activity

### 4.1. Absorption and Metabolism of Polyphenols in Gastrointestinal Tract

**Figure 1:**
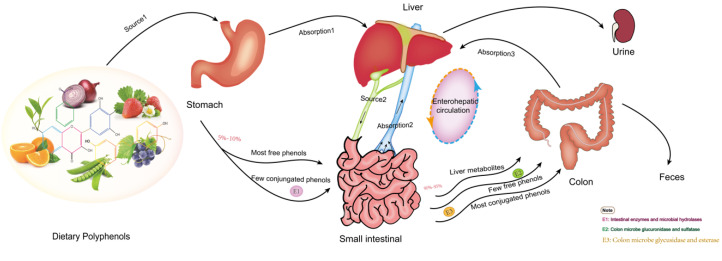
The transformation and absorption of polyphenols in the human body.

Studies have verified it is not natural polyphenols that ultimately make effects on cells and tissues, which is due to the transformation of their structure and activity in the process of absorption and utilization. Most dietary polyphenols exist in food as esters, glycosides or polymers, which cannot be used directly and must be absorbed after the action of gastrointestinal flora and enzymes. In the past, the biological community generally believed that polyphenols’ digestion and metabolism mainly occurred in the small intestine. However, recent research on the morphology of polyphenols and gastrointestinal organisms have led to a new understanding of their absorption and metabolism. Figure 1 shows the absorption and metabolism of the ingested polyphenols in the body. Dietary polyphenols are divided into free and conjugated polyphenols [72]. It is estimated that only 5–10% of free phenols with a simple structure, such as aglycons, flavonol monomers or dimers, and some polyphenol sugars, are absorbed by small intestine cells [73]. Some free phenols are transformed into metabolites available for resident microorganisms to produce biomass. These metabolites may even be more bioactive than their precursors. These simple phenols undergo phase I and II reactions in intestinal cells and hepatocytes, such as methylation, glucuronidation, and sulfated derivatives, to produce many water-soluble metabolites released into blood and various organs, and finally discharged from the urine. Almost 90–95% of dietary phenols cannot be absorbed by small intestine cells [74]. Conjugated phenols, such as oligomeric and polymerized phenols with a molecular weight of nearly 40 kDa, enter the colon. Only a few are absorbed by colon cells. Most participate in the catabolism of the intestinal flora, enter the enterohepatic circulation, and are finally absorbed and utilized by the human body, thus promoting health [75].

### 4.2. Intestinal Flora Regulates Biotransformation and Activity of Polyphenols

Intestinal flora has irreplaceable impacts on the complete metabolism of polyphenol glycosides. Polyphenols decompose into small-molecule metabolites absorbed and distributed in various tissues by intestinal microorganisms. Studies have reported that differences in intestinal flora affect biological functions of polyphenols [76]. The regulatory function of polyphenols is mainly reflected in the following aspects: (1) intestinal microorganisms secrete enzymes promote the transformation of polyphenols from conjugated to unconjugated [77]; (2) intestinal flora directly promotes the decomposition of free polyphenols into more active and easily absorbed molecules, which are absorbed into the intestinal liver circulation through intestinal mucosal cells; (3) through the depolymerization of intestinal microbial enzymes, phenol metabolites are excreted through the bile duct and some are reabsorbed; (4) through microbial metabolism, small phenolic metabolites with higher absorbability, utilization, and biological activity than precursor compounds, and some even have broader natural characteristics than original polyphenols [78]; (5) intestinal flora has specificity for the metabolic degradation of polyphenols. Different types of polyphenols can be absorbed by different flora. If there is no particular flora in the intestine, even if some polyphenols are ingested, they are not biologically active. Table 1 lists representative studies on the impact of the intestinal flora on polyphenol conversion and absorption.

### 4.3. Regulation of Polyphenols on the Intestinal Flora

The intestinal flora and the human body constitute the intestinal microecosystem and are closely related to health. The proportion of beneficial bacteria in the intestines of healthy people is 70%, whereas that of patients with cancer is only 10%. As shown in Table 2, studies including vitro fermentation models, animal models and clinical trials, have revealed polyphenols and metabolites’ regulatory functions on intestinal flora. Polyphenols selectively promote the proliferation of beneficial intestinal flora, inhibit pathogenic bacteria, reduce their virulence through prebiotic effects, regulate the composition of intestinal flora, enrich the diversity of intestinal flora, and promote intestinal microenvironment homeostasis [86]. The regulatory effect of polyphenols on gut microbiota might be affected to their structure, concentration, and microbial species. The reported mechanisms of polyphenols and intestinal flora are as follows: (1) polyphenol metabolites provide metabolic substrates for microbial growth and (2) polyphenols affect the activity of enzymes related to microbial growth. The specific impact may be as follows: (1) They affect the type and quantity of enzymes in the intestine by changing the type and content of gut microbiota and (2) chelating metal ions in the body. Some microbial enzyme systems with metal ions as coenzymes lose their activity due to the lack of auxiliary groups. Additionally, polyphenols and iron binding inhibit the heme group production in some aerobic microorganisms, which affects the microorganism and its enzymatic system. (3) They directly inhibit the activity of some intestinal microbial enzymes. For example, studies have shown that condensed tannins can inhibit the activity of bacterial extracellular enzymes, such as endoglucanase, which is mainly achieved by the combination of polyphenols and enzyme protein molecules. (4) There may be action on microbial cell membranes: the hydroxyl structure of polyphenols can combine with the bacterial cell membrane to inhibit bacteria and (5) influence microbial adhesion. For example, procyanidins B1 and B2 significantly increase the adhesion ability of Lactobacillus spp. In a word, polyphenols regulate intestinal flora, promote the growth of intestinal probiotics, inhibit pathogenic bacteria, inhibit carcinogenic enzymes activity in the microbiota, and reduce the probability of carcinogenesis.

## 5. Gut Microbiome and GC Treatment

The intestinal microbiome is closely related to GC, which influences the curative effect of different treatment strategies for GC, including surgery, chemotherapy, radiotherapy, and immunotherapy. Intestinal probiotics regulate the homeostasis of intestinal microbiota and maintain the intestinal barrier and immune state, which is beneficial to the recovery and improvement of post-operative prognosis [102]. In chemotherapy, intestinal flora can enhance efficacy, reduce drug resistance and reduce adverse events. Intestinal flora interaction promotes inflammation and provides inflammatory mediators for the treatment of oxaliplatin, cisplatin and CpG oligonucleotides. Research in mice has shown that antibiotic-treated mice (which killed the gut microbiome) did not respond as well to platinum chemotherapy or CpG-oligonucleotide immunotherapy as mice with intact gut microbes [103]. Other studies have reported that regulating the microbiome through nutrition or probiotic supplements can reduce chemotherapy toxicity and subsequent adverse events in mice and humans [104]. Additionally, research demonstrated that intestinal flora improves chemoresistance. For instance, Fusobacterium nucleatum regulating the molecular network of Toll-like receptors, microRNAs, and autophagy control the chemotherapy resistance of colorectal cancer clinically, biologically and mechanically [105]. Similarly, Fusobacterium nucleatum promotes chemoresistance of oxaliplatin by activating autophagy in tumor cells [106]. In radiotherapy, fecal flora transplantation (FMT) improves the survival rate of irradiated animals, gastrointestinal function, and intestinal epithelial integrity, and prevents radiation-induced toxicity. Moreover, intestinal microbiological disorders may become a potential biomarker for the prediction and prevention of radiation-induced bowel disease or other complications in the future [107]. In immunotherapy, the effect of intestinal microorganisms on the therapeutic efficacy and toxicity of immune checkpoint inhibitors (ICIs) has also been explored to a great extent [108]. Although the exact mechanism is unclear, Gopalakrishnan et al. showed that intestinal microflora remotely controls the central role of lymphocyte and myeloid cell regulation [109]. The release of lipopolysaccharide (LPS) from intestinal microorganisms stimulates innate immunity through TLR4 pathway, thus promoting anti-tumor CD8+ T cell immune response [110]. Certain bacteria, such as Bacteroidetes thetaiotaomicron and Faecali bacterium prausnitzii, have been reported to enhance the effectiveness of checkpoint inhibitors [111]. *Hp* is recognized as a pathogenic factor in gastric cancer, but recently, researchers have found that *Hp* influences gastric cancer immunotherapy. Liu et al. demonstrated that 59.3% of *Hp+* GC patients expressed PD-L1, suggesting that *Hp* might imply anti-PD-1/PD-L1 therapy efficacy [112]. Wu et al. proved that PD-L1 expression in primary human gastric epithelial cells is strongly enhanced by *Hp*, and significantly induces T cell apoptosis to enhance the efficacy of immune checkpoint inhibitors [113]. Finally, in a recent study (DELIVER test: UMIN000030850), Bacterial genome analysis of 501 patients with advanced GC treated with nivolumab showed that Odoribacter and Veillonella were associated with tumor response to nivolumab, and GC-specific intestinal microflora may predict the response to ICIs [114]. In all, the role of gastrointestinal microflora in GC treatment needs to be further clarified in multicenter prospective studies to identify specific bacterial species and pathways, as well as changes in microbiota associated with the progression of GC.

## 6. Summary and Challenge

As natural plant compounds, dietary polyphenols have great potential for chemical prevention and therapy of GC. Polyphenols are anti-inflammatory, antibacterial, antioxidant, and anti-proliferative compounds that induce apoptosis or autophagy, inhibit EMT, cause the hindering of angiogenesis and metastasis, enhance chemotherapy sensitivity, and regulate gastrointestinal flora to play a protective role against GC [41,115,116].

Currently, although much progress has been made in understanding the anti-GC mechanism related to polyphenols, the details are still unclear as to how dietary polyphenols affect these mechanisms. Polyphenols act as localized small-molecule inhibitors in signal transduction and block their protein–protein interactions or their interactions with DNA, in particular the disruption of multimeric forms of transcription factors such as c-jun/c-fos (Activator Protein-1; AP-1) [117], c-myc/max, nuclear factor kappa-light-chain-enhancer of activated B cells (NF-κB) [118] and β-catenin/T cell factor (Tcf), thus having an anti-tumor effect. The development of polyphenol drugs, such as polyphenol transcription factor inhibitors, has significant clinical application value [119]. Additionally, as mentioned above, human topoisomerase may serve as a potential molecular target of polyphenol compounds, which can inhibit enzyme activity and ultimately prevent the growth of cancer cells [120]. In the future, we can develop polyphenol compounds as cancer cell topoisomerase inhibitors, providing more possibilities for anticancer drugs. However, polyphenols, as natural compounds, have low bioavailability in our bodies. More attention should be paid to how to deliver higher concentrations of polyphenols to target organs to improve their absorption and utilization. The existing polyphenol nano-drug delivery technology may have great potential in this regard [121]. In fact, studies have demonstrated that polyphenols can provide a powerful environment for tumor immunotherapy by regulating the tumor immune microenvironment (TME) [122]. Conversely, there are indications that polyphenols may also play harmful roles [123], which means we should choose carefully when immunotherapy is used [124]. Future studies focused on precise immunotherapeutic protocols and well-defined cell and animal models will probably help us explore new ways to fight cancer. Importantly, studies have reported that the polyphenol compound naringin cannot be hydrolyzed by rhamnosidase in probiotics but can be hydrolyzed by fungal rhamnosidases, which indicates that intestinal fungi have a specific effect on the catabolism of polyphenols [79]. Similarly, studies have reported the effect of polyphenols on fungi [125], but the interaction mechanism between polyphenols and fungi still needs to be further studied. What is the interaction between intestinal fungi and intestinal bacteria in the anti-GC activity of polyphenols? What are the potential connections between polyphenols, intestinal bacteria, and intestinal fungi? In the future, more basic and clinical research will be required to understand the interaction mechanisms among polyphenols, GC and other influencing factors.

## Figures and Tables

**Table 1:** The effect of intestinal flora on transformation and absorption of polyphenol.

Research Type	Polyphenol	Effects of Intestinal Flora on Polyphenols	Reference
In vitro	Flavonoid	Probiotic rhamnosidase promotes hydrolysis of hesperidin and Narcissus, but Naringin only is hydrolyzed by fungal rhamnosidase.	[79]
In vitro	Flavonoid	Intestinal flora helps Formonoside produce two metabolites (6′-o-malonyl Formonoside, 6′-o-malonyl).	[80]
In vitro	Flavonoid	Escherichia coli converts daidzein into equol by microbial enzymes (ORF-1 enzyme, ORF-2 enzyme, ORF-3 enzyme).	[81]
In vivo	Querceti	Plasma quercetin metabolites concentration is positively correlated with Enterobacteriaceae count.	[82]
In vivo	Procyanidine	Lactobacillus Casei-01 transforms procyanidine into 3-o-flavan and improves its antioxidant capacity.	[83]
In vivo and In vitro	Lignans (SDG)	SDG is deglycosylated to ring-opening isolarch oleoresin (SECO); matairesinol and anhydrosecoi-solariciresinol (AHS) are new intermediates.	[84]
In vivo and In vitro	Trans-Resveratrol	Slackia Equolifaciens and Adlercreutzia Equolifaciens transforms dihydroresveratrol into new trans-resveratrol metabolites (3,4’-dihydroxy-trans-stilbene, 3,4’-dihydroxy-biphenyl).	[85]

**Table 2:** The regulation of polyphenols and polyphenol-rich extracts on intestinal flora.

Study Type	Polyphenol	Regulation of Polyphenols on Intestinal Flora	Reference
In vitro	Cocoa phenol	Increases production of Bifidobacteria and Lactobacillus.	[87]
In vitro	Areca seed polyphenol	Increases intestinal flora species diversity and changes Proteobacteria and Firmicutes relative abundance ratio.	[88]
In vitro	Tea polyphenol	Improves Bacteroidetes and Firmicutes relative abundance and reduces their ratio.	[89]
Animal experiment	Cocoa phenol	Decreases Bacteroides, Clostridium, and Staphylococcus proportion.	[90]
Animal experiment	Pomegranate phenol	Reduces inflammatory markers (iNOS, cyclooxygenase-2, ptges, and PGE-2).	[91]
Animal experiment	Apple polyphenol	Promotes Lactobacillus and Bifidobacterium.	[92]
Animal experiment (mouse)	Cranberry polyphenol	Increases intestinal mucin degrading bacteria (Akkermansia muciniphila).	[93]
Animal experiment (mouse)	Grape polyphenol	Increases Akkermansia Muciniphila and decreases Firmicutes and Bacteroidetes.	[94]
Animal experiment (mouse)	Resveratrol	Inhibites Enterococcus faecalis, and promotes Lactobacillus and Bifidobacteriums.	[95]
Animal experiment (mouse)	Tea polyphenol	Increases Bacteroidetes and Proteus and decreases Firmicutes.	[96]
Animal experiment (mouse)	Tea polyphenol	Promotes Bifidobacterium.	[97]
Animal experiment (mouse)	Grape-seed polyphenol	Promotes Lactobacillus, Bacteroides and Bifidobacterium, inhibites Barnes, Ehrlich Shigella and Ekman.	[98]
Clinical study (RCT)	Red wine polyphenol	Promotes Enterococcus, Prevotellas, Bacteroides, Bifidobacteria, Bacteroides, and Eubacterium.	[99]
Clinical study (RCT)	Total polyphenols	Increases fiber fermentation and butyrate-producing bacterias.	[100]
Clinical study (RCT)	hesperidin and naringin	Increases the production of short-chain fatty acids, and reduces ammonia nitrogen.	[101]
